# Role of Terlipressin in Cirrhotic Patients with Ascites and without Hepatorenal Syndrome: A Systematic Review of Current Evidence

**DOI:** 10.1155/2020/5106958

**Published:** 2020-06-22

**Authors:** Zhaohui Bai, Yang An, Xiaozhong Guo, Rolf Teschke, Nahum Méndez-Sánchez, Hongyu Li, Xingshun Qi

**Affiliations:** ^1^Department of Gastroenterology, General Hospital of Northern Theater Command, Shenyang 110840, China; ^2^Postgraduate College, Shenyang Pharmaceutical University, Shenyang 110840, China; ^3^Department of Internal Medicine II, Division of Gastroenterology and Hepatology, Klinikum Hanau, D-63450 Hanau, Germany; ^4^Liver Research Unit, Medica Sur Clinic and Foundation and Faculty of Medicine, National Autonomous University of Mexico, Mexico City, Mexico

## Abstract

Ascites, a common complication in cirrhosis, is prone to the development of acute kidney injury or hepatorenal syndrome and can be complicated by circulatory dysfunction after paracentesis. Terlipressin has not been considered as the mainstay treatment option for ascites in cirrhosis yet. The present work aimed to systematically review the current evidence regarding the use of terlipressin in cirrhosis with ascites and without hepatorenal syndrome. PubMed, EMBASE, and Cochrane Library databases were searched for relevant studies. Twelve studies were eligible. In 3 studies (1 randomized controlled trial and 2 single-arm studies without controls) involving 32 patients who received terlipressin for nonrefractory ascites, terlipressin improved hemodynamics by decreasing the heart rate and cardiac output and increasing the mean arterial pressure and systemic vascular resistance. In 5 studies (1 randomized controlled trial, 2 single-arm studies without controls, and 2 comparative studies with controls) involving 67 patients who received terlipressin for refractory ascites, terlipressin improved renal function by increasing the glomerular filtration rate, renal blood flow, urinary sodium, and urine output and decreasing serum creatinine. In 4 studies (4 randomized controlled trials) involving 71 patients who received terlipressin for preventing from paracentesis-induced circulatory dysfunction, terlipressin prevented from paracentesis-induced circulatory dysfunction by increasing the mean arterial pressure and systemic vascular resistance and decreasing plasma renin. Terlipressin may improve hemodynamics, severity of ascites, and renal function and prevent from paracentesis-induced circulatory dysfunction in cirrhosis with ascites and without hepatorenal syndrome. However, no study has evaluated the effect of terlipressin for prevention of acute kidney injury.

## 1. Introduction

Ascites, a serious complication of cirrhosis [1], is secondary to the activation of endogenous sodium and water retention systems and visceral vasodilation [2], which has a 1- and 5-year survival rate of 85% and 57%, respectively [3]. Current treatment of cirrhotic ascites mainly includes sodium limitation, diuretics, paracentesis, supplementation of human albumin, and transjugular intrahepatic portosystemic shunt (TIPS) [1]. On the contrary, the risk of renal failure is increased in cirrhosis with ascites [4, 5]. Its potential mechanism is that splanchnic vasodilatation can reduce the arterial blood volume and pressure and then activate vasoconstrictors and the antinatriuretic factors, thereby resulting in retention of sodium and water, impairment of renal excretion of free water, and renal vasoconstriction [2, 6]. Terlipressin reduces portal inflow through direct and potent vasoconstriction of splanchnic vessels [7] and improves the hyperdynamic state [8]. It seems that terlipressin should be an effective adjunctive choice of therapy for improving ascites and preventing renal dysfunction in cirrhosis with ascites [9]. However, until now, no recommendation has been given about the use of terlipressin in cirrhotic patients with ascites and without hepatorenal syndrome. Herein, we performed a systemic review to explore this issue.

## 2. Methods

### 2.1. Registration

This work was registered in PROSPERO.

### 2.2. Search Strategy and Study Selection

We retrieved all papers via the PubMed, EMBASE, and Cochrane Library databases. The time interval was from the earliest available publication until August 30, 2019. The following keywords were used: (Terlipressin) AND (Cirrhosis) AND (Ascites). All eligible papers explored the role of terlipressin in cirrhosis with ascites. Exclusion criteria were as follows: (1) duplicates; (2) case reports, comments, or letters; (3) guidelines, reviews, or meta-analyses; (4) animal studies; (5) patients did not have cirrhosis; (6) ascites was not evaluated in cirrhosis; (7) patients were not treated by terlipressin; (8) trial registration; and (9) full texts cannot be obtained.

### 2.3. Data Collection

The following information was extracted from each study: first author, year of publication, country, study design, type of study, study duration, source of cases, number of patients with cirrhosis and ascites, number of patients treated with and without terlipressin, weight, urine volume, urinary sodium, urinary sodium excretion, cardiac index, heart rate, systemic vascular resistance, mean arterial pressure, hepatic venous pressure gradient, sodium, and/or serum creatinine.

### 2.4. Groups

Terlipressin group should be that patients received terlipressin infusion. If any, the control group should be that patients received standard medical treatment without additional terlipressin infusion.

### 2.5. Outcomes

Outcomes mainly included dynamic change of hemodynamic parameters, improvement of ascites, change of renal function parameters, and risk of hepatorenal syndrome or acute kidney injury, if any.

### 2.6. Study Quality Assessment

We used the Newcastle–Ottawa Scale (NOS) for assessing the quality of nonrandomized studies, which includes selection, comparability, exposure, and outcome. The NOS uses a semiquantitative star-level system to evaluate the quality of studies. The quality of included randomized controlled trial studies will be evaluated using the Cochrane Collaboration's tool for assessing the risk of bias.

## 3. Results

### 3.1. Characteristics of Studies

**Figure 1:**
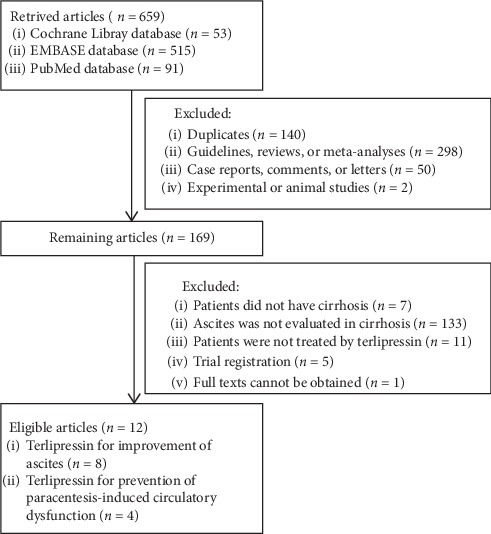
Flowchart of study selection.

**Figure 2:**
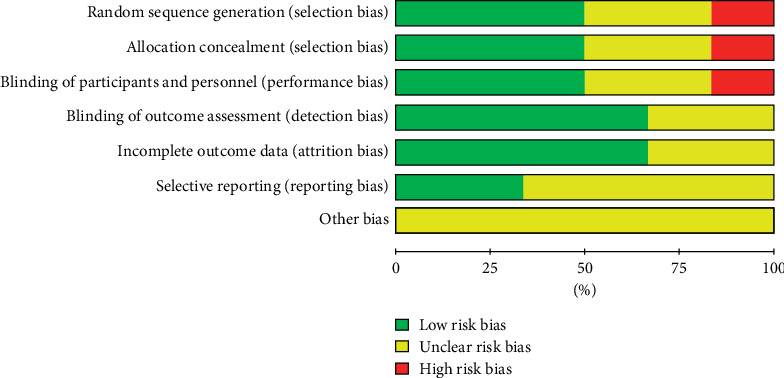
Quality of included randomized controlled trials.

We identified 659 studies through the PubMed, EMBASE, and the Cochrane Library databases. Finally, 12 studies were included (Figure 1). The characteristics of the included studies are listed in Table 1. The sample size ranged from 5 to 26 cases. The included studies were published between 1997 and 2016. Eleven studies were published as full texts, and the remaining study was published as an abstract. According to the regions, two studies were performed each in Greece, France, India and one study each in UK, Italy, Denmark, Egypt, Australia, and Czech Republic. Among them, 8 studies aimed to explore the role of terlipressin in the improvement of ascites in cirrhosis and 4 studies aimed to explore the role of terlipressin in the prevention of paracentesis-induced circulatory dysfunction in cirrhosis. Five studies were randomized controlled trials. The quality of these included randomized controlled trials is summarized in Figure 2. Four studies were single-arm studies without control groups, and 3 studies were comparative studies with control groups. The quality of these nonrandomized studies is summarized in Table 2.

### 3.2. Use of Terlipressin in Cirrhosis with Nonrefractory Ascites

In 2004, Therapondos et al. [8] reported self-control data regarding the use of terlipressin in 6 cirrhotic patients with moderate-severe ascites who were treated with terlipressin. They found that heart rate and cardiac output were significantly decreased and mean arterial pressure and systemic vascular resistance were significantly increased, whereas renal blood flow, urinary volume, and urinary sodium excretion remained virtually unchanged.

In 2007, Krag et al. [9] randomly assigned 11 and 4 patients with nonrefractory ascites into terlipressin and no terlipressin groups, respectively. As compared with patients who did not receive terlipressin, patients who received terlipressin had a greater improvement of the glomerular filtration rate, sodium clearance, urine sodium concentration, plasma norepinephrine, and plasma renin.

In 2010, Kalambokis et al. [10] reported self-control data regarding the use of terlipressin in 15 cirrhotic patients with ascites that was not massive, tense, or refractory. They found that terlipressin significantly increases the water excretion, creatinine clearance, sodium excretion, mean arterial pressure, and systemic vascular resistance and significantly decreases cardiac output.

### 3.3. Use of Terlipressin in Cirrhosis with Refractory Ascites

In 1997, Gadano et al. [11] reported self-control data regarding the use of terlipressin in 8 cirrhotic patients with refractory ascites. They found that terlipressin significantly decreases the hepatic venous pressure gradient, cardiac index, and heart rate and significantly increases the mean arterial pressure, systemic vascular resistance, and urinary sodium excretion.

In 2007, Krag et al. [9] reported self-control data regarding the use of terlipressin in 8 cirrhotic patients with refractory ascites. They found that terlipressin significantly increases the renal blood flow, glomerular filtration rate, sodium clearance, and urine sodium concentration and significantly decreases the plasma norepinephrine and renin.

In 2011, Fimiani et al. [12] reported self-control data regarding the use of terlipressin in 26 cirrhotic patients with refractory ascites. Among them, 16 patients had complete response after treatment of terlipressin, which was defined as a reduction of abdominal circumference of approximately 10%, a reduction of body weight of at least 3 kg per week, and a four-fold increase of urinary sodium excretion.

In 2016, Gow et al. [14] reported self-control data regarding the use of terlipressin in 5 outpatients with cirrhosis and refractory ascites. All of them had a statistically significant decrease in body weight and ascites volume and a statistically significant increase in urine sodium excretion. Serum creatinine was slightly decreased, but the difference was not statistically significant.

In 2016, Pande et al. [19] published an abstract in which 20 and 25 patients with refractory ascites were treated with or without terlipressin, respectively. They reported self-control data alone but did not compare the difference between terlipressin and no terlipressin groups. The absolute serum creatinine and body weight were decreased and the absolute urinary sodium excretion and urine output were increased in the terlipressin group.

### 3.4. Use of Terlipressin in Cirrhosis with Unclassified Ascites

In 2005, Kalambokis et al. [13] reported self-control data regarding the use of terlipressin in 11 cirrhotic patients with unclassified ascites and esophageal varices. They found that terlipressin significantly decreases the portal vein velocity, portal flow volume, and cardiac output and increases the mean arterial pressure and systemic vascular resistance. However, sodium excretion remained virtually unchanged.

### 3.5. Use of Terlipressin for Paracentesis-Induced Circulatory Dysfunction in Cirrhosis with Tense Ascites

Three head-to-head randomized controlled trials [15–17] compared the effects of terlipressin versus albumin infusion for preventing from paracentesis-induced circulatory dysfunction. All of them showed that terlipressin was comparable to albumin for the prevention of paracentesis-induced circulatory dysfunction in cirrhosis with tense ascites.

One head-to-head randomized controlled trial by Abdullah et al. [18] compared the effects of terlipressin versus norepinephrine for preventing paracentesis-induced refractory hypotension. Both terlipressin and norepinephrine successfully prevented from postparacentesis refractory hypotension and decrease of systemic vascular resistance. Terlipressin, rather than norepinephrine, had a renal protective effect during the postoperative period.

## 4. Discussion

**Figure 3:**
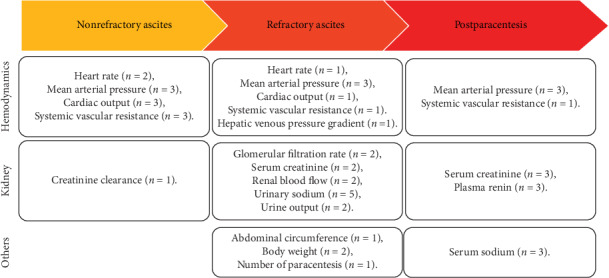
An overview of findings regarding efficacy of terlipressin in nonrefractory studies, refractory ascites, and paracentesis-induced circulatory dysfunction population.

This is the first systematic review of the literature to explore the role of terlipressin on cirrhotic patients with ascites and without hepatorenal syndrome. Despite relevant studies were extensively searched, only 12 studies regarding the role of terlipressin in cirrhosis with ascites were included in this systemic review. In the current work, according to the goal of terlipressin among studies, we divided the study population into refractory ascites, nonrefractory studies, unclassified ascites, and paracentesis-induced circulatory dysfunction groups. Terlipressin, a vasopressin derivative with enhanced vasoconstrictive properties, has been widely used in cirrhotic patients with variceal bleeding or hepatorenal syndrome [20, 21]. In our systemic review, terlipressin likely improves the hemodynamics, severity of ascites, and renal function in cirrhotic patients with ascites (Figure 3).

When the portal pressure is gradually increased, collateral veins develop and vasodilators are produced, thereby leading to splanchnic arterial vasodilatation. Then, fluids collect in the peritoneal cavity with a decrease in central blood volume [22–24]. Our systemic review suggests the beneficial effect of terlipressin on hemodynamics in nonrefractory ascites, including an increase in mean arterial pressure and systemic vascular resistance and a decrease in heart rate and cardiac output, thereby increasing central blood volume.

When ascites is further aggravated, the renin-angiotensin-aldosterone system (RAAS) is significantly activated, resulting in renal sodium and water retention, which is necessary to replenish the intravascular volume in order to maintain hemodynamic stability [25]. In refractory ascites, renal sodium retention becomes more severe and renal perfusion and glomerular filtration rate further decrease, following the progression of systemic and portal hemodynamic abnormalities [26, 27], thereby leading to renal function impairment. Our systemic review suggests that terlipressin significantly improves the glomerular filtration rate, serum creatinine, renal blood flow, urinary sodium, and urine output, except for hemodynamics.

Paracentesis can rapidly reduce the circulating blood volume after transfer of massive fluids [28] and then activate the sympathetic nervous system and RAAS and increase the plasma renin activity, thereby inducing circulatory dysfunction [29, 30]. On the contrary, cardiac output is significantly increased after paracentesis [31], and then nitric oxide synthesis is elevated, which decreases systemic vascular resistance, thereby resulting in circulatory dysfunction [32]. Our systemic review suggests that terlipressin is potentially advantageous for preventing from paracentesis-induced circulatory dysfunction by increasing the mean arterial pressure and systemic vascular resistance and decreasing the plasma renin.

A major limitation is the heterogeneity in the severity of ascites and treatment approaches for ascites among studies although we analyzed the studies according to the different disease conditions. Thus, we did not perform any meta-analysis to synthesize the data. Additionally, the quality of the current evidence is relatively low, which is mainly based on small sample size single-arm observational studies. Few studies focused on hard endpoints, such as the risk of acute kidney injury and hepatorenal syndrome and death.

Based on the present systematic review of the literature, terlipressin may be beneficial in cirrhosis with ascites and without hepatorenal syndrome. However, the efficacy of terlipressin for preventing from acute kidney injury in cirrhotic patients with ascites should be further explored by large-scale observational studies or randomized controlled trials. Meanwhile, adverse reactions of terlipressin, which mainly include headache, hypertension, vomiting, stomachache, and hyponatremia, should be evaluated in such patients.

## Figures and Tables

**Table 1:** Characteristics of included studies.

First author (year)	Country	Characteristics of patients	No. pts	Groups	Dose of terlipressin	Other treatment of ascites	Main outcomes
*Nonrefractory ascites*							
Therapondos (2004) [8]	UK	Cirrhosis with moderate-severe ascites	6	Terlipressin: 6	2 mg Single dosage	Diuretic	HR, MAP, CO, SVR, HVPG, RBF, urinary flow, Na
Kalambokis (2010) [10]	Greece	Cirrhosis with ascites without hyponatremia	15	Terlipressin: 15	2 mg Single dosage	Diuretic	Water excretion, MAP, CO, SVR, creatinine clearance

*Refractory ascites*							
Gadano (1997) [11]	France	Cirrhosis with refractory ascites	16	Terlipressin: 8	1-2 mg Single dosage	Low-sodium diet	Natriuretic response, MAP, RBF, GFR, SCr
				Control: 8	None	Low-sodium diet	
Krag (2007) [9]	Denmark	Cirrhosis with refractory or nonrefractory ascites	23	Terlipressin: 19	2 mg Single dosage	Low-sodium diet	MAP, RBF, GFR, SCr
				Placebo: 4	None	Low-sodium diet	
Fimiani (2011) [12]	Italy	Cirrhosis with refractory ascites	26	Terlipressin: 26	0.5 mg/6 h–1 mg/6 h for 3 weeks	Diuretic + albumin	Response of refractory ascites, body weight, urinary sodium excretion
Pande (2016) [13]	India	Cirrhotic patients with refractory ascites	45	Terlipressin: 20	4 mg/12 h during hospitalizations	Diuretic + albumin	Na, SCr, body weight, urinary sodium
				Control: 25	None	Diuretic + albumin	
Gow (2016) [14]	Australia	Cirrhosis with diuretic refractory ascites	5	Terlipressin: 5	3.4 mg/24 h for 28 days	Paracentesis	Response of refractory ascites, body weight, urinary sodium excretion, SCr, MAP

*Unclassified ascites*							
Kalambokis (2005) [13]	Greece	Cirrhosis with ascites	11	Terlipressin: 11	2 mg Single dosage	Somatostatin	Urine urea, SCr, Na

*Paracentesis-induced circulatory dysfunction*							
Moreau (2002) [15]	France	Cirrhosis with tense ascites	20	Terlipressin: 10	3 mg Single dosage	Paracentesis	Arterial blood volume, plasma renin concentrations, plasma aldosterone concentrations, SCr, Na, MAP, urinary sodium excretion
				Albumin: 10	None	Paracentesis	
Singh (2006) [16]	India	Cirrhosis with tense ascites	40	Terlipressin: 20	1 mg in 0 h, 8 h, and 16 h after paracentesis	Paracentesis	MAP, Na, SCr, plasma renin activity
				Albumin: 20	None	Paracentesis	
Lata (2007) [17]	Czech Republic	Cirrhosis with tense ascites	49	Terlipressin: 24	1 mg/4 h for 48 h	Paracentesis	Blood pressure, HR, diuresis, ECG, Na, plasma renin activity
				Albumin: 25	None	Paracentesis	
Abdullah (2012) [18]	Egypt	Cirrhosis with tense ascites	34	Terlipressin: 17	After single dosage of 1 mg/30 min followed 2 *μ*g/kg/h	Paracentesis	HR, MAP, SVR, total bilirubin, urine output
				Norepinephrine: 17	None	Paracentesis	

Abbreviations: pts, patients; HR: heart rate; MAP: mean arterial pressure; Na: serum sodium; SCr: serum creatinine GFR: glomerular filtration rate; CO: cardiac output; SVR: systemic vascular resistance; HVPG: hepatic venous pressure gradient; RBF: renal blood flow; ECG: electrocardiograph; NA: not applicable.

**Table 2:** Quality of nonrandomized studies.

First author (year)	Selection				Comparability	Outcome			Total
	Q1	Q2	Q3	Q4	Q5	Q6	Q7	Q8	
Therapondos (2004) [8]	—	—	^*∗*^	^*∗*^	—	^*∗*^	^*∗*^	^*∗*^	5
Kalambokis (2010) [10]	—	—	^*∗*^	^*∗*^	—	^*∗*^	^*∗*^	^*∗*^	5
Fimiani (2011) [12]	—	—	^*∗*^	^*∗*^	—	^*∗*^	^*∗*^	^*∗*^	5
Abdullah (2012) [18]	—	^*∗*^	^*∗*^	^*∗*^	^*∗*^	^*∗*^	^*∗*^	^*∗*^	7
Gow (2016) [14]	—	—	^*∗*^	^*∗*^	—	^*∗*^	^*∗*^	^*∗*^	5
Pande (2016) [19]	—	^*∗*^	^*∗*^	^*∗*^	^*∗*^	^*∗*^	^*∗*^	—	6

Notes: Q1: representativeness of the exposed cohort; Q2: selection of the nonexposed cohort; Q3: ascertainment of exposure; Q4: demonstration that outcome of interest was not present at the start of the study; Q5: comparability of cohorts on the basis of the design or analysis; Q6: assessment of the outcome; Q7: was follow-up long enough for outcomes to occur; Q8: adequacy of follow-up of cohorts.

## Data Availability

This is a review paper without any original data involved.
